# Activation of STAT and SMAD Signaling Induces Hepcidin Re-Expression as a Therapeutic Target for β-Thalassemia Patients

**DOI:** 10.3390/biomedicines10010189

**Published:** 2022-01-17

**Authors:** Hanan Kamel M. Saad, Alawiyah Awang Abd Rahman, Azly Sumanty Ab Ghani, Wan Rohani Wan Taib, Imilia Ismail, Muhammad Farid Johan, Abdullah Saleh Al-Wajeeh, Hamid Ali Nagi Al-Jamal

**Affiliations:** 1 School of Biomedicine, Faculty of Health Sciences, Universiti Sultan Zainal Abidin, Kuala Nerus 21300, Terengganu, Malaysia; si2751@putra.unisza.edu.my (H.K.M.S.); wanrohani@unisza.edu.my (W.R.W.T.); imilia@unisza.edu.my (I.I.); 2 Pathology Department, Hospital Sultanah Nur Zahirah, Kuala Terengganu 20400, Terengganu, Malaysia; dralawiyah@moh.gov.my (A.A.A.R.); drazlysumanty@moh.gov.my (A.S.A.G.); 3 Department of Haematology, School of Medical Sciences, Universiti Sains Malaysia, Kubang Kerian 16150, Kelatan, Malaysia; faridjohan@usm.my; 4 Anti-Doping Lab Qatar, Doha 27775, Qatar; asaleh@adlqatar.qa

**Keywords:** *hepcidin*, HbE/β-thalassemia, iron overload, ferroportin, iron homeostasis, signaling pathways

## Abstract

Iron homeostasis is regulated by *hepcidin*, a hepatic hormone that controls dietary iron absorption and plasma iron concentration. *Hepcidin* binds to the only known iron export protein, ferroportin (FPN), which regulates its expression. The major factors that implicate *hepcidin* regulation include iron stores, hypoxia, inflammation, and erythropoiesis. When erythropoietic activity is suppressed, *hepcidin* expression is hampered, leading to deficiency, thus causing an iron overload in iron-loading anemia, such as β-thalassemia. Iron overload is the principal cause of mortality and morbidity in β-thalassemia patients with or without blood transfusion dependence. In the case of thalassemia major, the primary cause of iron overload is blood transfusion. In contrast, iron overload is attributed to hepcidin deficiency and hyperabsorption of dietary iron in non-transfusion thalassemia. Beta-thalassemia patients showed marked *hepcidin* suppression, anemia, iron overload, and ineffective erythropoiesis (IE). Recent molecular research has prompted the discovery of new diagnostic markers and therapeutic targets for several diseases, including β-thalassemia. In this review, signal transducers and activators of transcription (STAT) and SMAD (structurally similar to the small mothers against decapentaplegic in Drosophila) pathways and their effects on *hepcidin* expression have been discussed as a therapeutic target for β-thalassemia patients. Therefore, re-expression of *hepcidin* could be a therapeutic target in the management of thalassemia patients. Data from 65 relevant published experimental articles on *hepcidin* and β-thalassemia between January 2016 and May 2021 were retrieved by using PubMed and Google Scholar search engines. Published articles in any language other than English, review articles, books, or book chapters were excluded.

## 1. Thalassemia Syndrome

Thalassemia is an inherited autosomal recessive blood disorder that can be divided into either alpha (α) or beta (β) depending on the affected α or β globin chain [1]. The adult hemoglobin (HbA) consists of two α and two β (α2β2) chains in each HbA molecule [2,3]. Alpha-thalassemia occurs if one or more of the four alleles that code for α globin is missing or damaged [4]. On the other hand, β-thalassemia is caused by mutation in the β globin gene leading to a reduction in β globin or production of abnormal hemoglobin.

## 2. Beta-Thalassemia

Notably, β-thalassemia is highly reported in the Mediterranean and Southeast Asian countries as one of the most common genetic disorders [5]. Nevertheless, a lack of information on knowledge, attitudes, and practices towards β-thalassemia poses a challenge in many countries, including Malaysia [5]. This disease can be categorized into β-thalassemia major, intermedia, or minor [6]. β-thalassemia major and intermedia are genetically homozygous or heterozygous (β^0^ and β^+^), whereas β-thalassemia minor is usually heterozygous [6].

The β globin chain is encoded by the β globin gene *(HBB)*, located on chromosome 11 at the short arm position 15.4 [7,8]. Individuals with β-thalassemia major and intermedia inherit the mutation in both copies of the *HBB* gene, affecting normal β globin chain production [7]. The clinical features of β-thalassemia major are revealed as early as the first two years of the patient’s life and are usually connected to transfusional iron overload, whereas the clinical presentation for β-thalassemia intermedia occurs later in life [2]. In contrast, β-thalassemia minor or trait has one mutation in the HBB gene and is described as a carrier [6]. They are usually symptomless, with a hypochromic microcytic blood picture and mild anemia, and can potentially increase in severity with malnutrition [6].

Beta-thalassemia can be present alongside other diseases associated with an abnormal β globin chain, such as the hemoglobin E (HbE) disease, exhibiting severe anemia [2]. HbE is a hemoglobin (Hb) variant caused by a single base substitution of glutamic acid to lysine at position 26 of the globin chain, commonly found in Southeast Asia [9]. It can be classified into three types: heterozygous, homozygous, or compound heterozygous [9]. When the HbE trait is coinherited with β-thalassemia, it is called compound heterozygous, a condition known as HbE/β-thalassemia, which resembles homozygous β^0^-thalassemia clinically and hematologically [10].

The phenotypic heterogeneity of HbE/β-thalassemia can range from mild asymptomatic anemia to a severe form that requires regular blood transfusion [11]. A study in Sri Lanka revealed that the HbE/β-thalassemia phenotype is unstable during the first 10 years of life but gradually stabilizes as the patient gets older [11]. This condition is caused by various changes in anemia and erythroid expansion progression during their early life [11]. However, the lack of knowledge on the classification of the disease severity will affect the understanding of HbE/β-thalassemia clinical progression with age [11].

## 3. Iron

**Figure 1:**
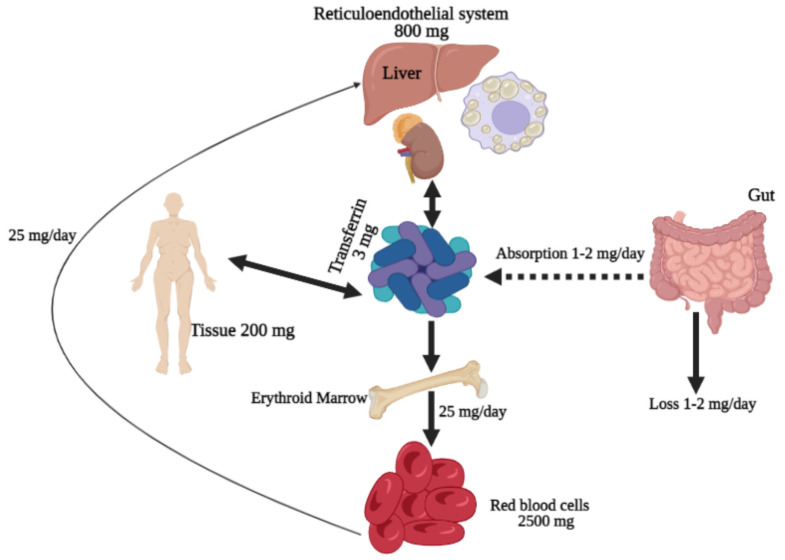
Iron distribution in the human body. Iron is absorbed by the duodenum enterocytes and into the plasma, where transferrin delivers it to bone marrow for Hb synthesis by erythroid precursors and erythrocytes or to muscles for myoglobin synthesis. Excess iron in the blood circulation can be stored as ferritin molecules in the liver or macrophages. The regular daily iron loss (1–2 mg) occurs mainly through blood loss (hemorrhage or menstruation).

Iron is an essential trace element found abundantly in the environment but is not readily available for uptake since it oxidizes when in contact with oxygen, thus making it highly insoluble [12]. The majority of iron in the body is found in red blood cells and is used to produce Hb during erythropoiesis [13,14]. In addition, trace amounts of iron are bound to enzyme effectiveness, such as cytochromes and those involved in the Krebs cycle [12]. Besides, iron can exist as non-hem compounds, such as ferritin and hemosiderin, primarily found in the spleen, bone marrow, and liver (see Figure 1) [12]. 

Iron in the form of ferrous ion (Fe^2+^) is an essential component of the Hb that binds with oxygen from the lungs before being transported to other parts of the body [15]. Furthermore, iron bound to cytochromes is vital for the electron transfer chain. It is reversibly recycled in the form of Fe^2+^ and ferric ion (Fe^3+^), aiding in energy production in the form of adenosine triphosphate [12]. On top of that, iron bound to peroxidases can convert potentially harmful hydrogen peroxide to water [16,17]. Other iron functions include deoxyribonucleic acid (DNA) replication, DNA repair, and cell signaling [15].

### 3.1. Iron Metabolism

The primary iron absorption takes place in the intestine, a crucial process in regulating the iron level in the body [18]. Iron is internalized as Fe^2+^ by divalent metal transporter 1 (DMT1) [19]. Initially, iron in food is in the form of Fe^3+^ and must undergo reduction via agents resembling vitamin C before it can be successfully absorbed [20,21]. Later, Fe^2+^ is bound by apoferritin in the intestinal cells, where it undergoes subsequent oxidation by extracellular protein, ceruloplasmin, to form Fe^3+^, which is bound by ferritin [22]. After that, apotransferrin helps iron absorption into the blood, where apotransferrin turns into transferrin by binding to two Fe^3+^ [23]. Transferrin carries iron in the plasma and releases it to organs such as bone marrow, where red blood cells are produced [24]. After about four months, iron is recycled from senescent red blood cells by the spleen, liver, and macrophages, ready to be reused [25]. The conservation of iron is critical because dietary iron is just enough to replace small losses [25]. Mice with iron overload were able to eliminate a significant amount of iron through the digestive tract by an unknown mechanism [26]. Most lost iron can be found in the epithelial and red blood cells excreted in the urine or feces [27]. An adult male is known to lose an average of 1 mg of iron per day, while women lose 1.4 mg per day through the menstrual cycle [28,29].

### 3.2. Iron Overload in β-Thalassemia

**Figure 2:**
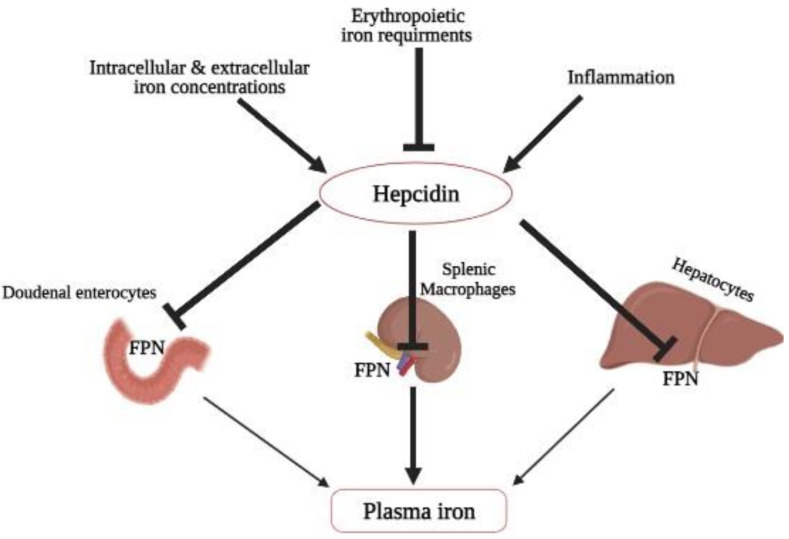
Hepcidin regulation on iron homeostasis: *hepcidin* synthesis is regulated at the transcriptional level by multiple stimuli. *Hepcidin* transcription increased with rising intra–extracellular iron concentrations and inflammation. In contrast, hepcidin production is suppressed in response to higher erythropoietic activity. Iron concentration in plasma is regulated by *hepcidin* through controlling *FPN* concentrations in iron exporting cells (duodenal, enterocytes, hepatocytes, and macrophages from liver and spleen). **➔**: resulting in or enhances expression and **⊥:** reduced expression.

The liver contains approximately 70% of the body iron, thus being the most affected organ during iron overload [30]. Hepcidin discovery as a key regulator of iron metabolism is revolutionary in understanding the mechanism of iron overload in β-thalassemia patients [31,32]. It is a peptide hormone first isolated from human urine and encoded by the *hepcidin* antimicrobial peptide *(HAMP)* gene [25]. When there is an increase in iron level in the body, the hepatocytes are stimulated to release more hepcidin into the bloodstream [33]. *Hepcidin* is also a crucial negative regulator of iron absorption into the body through the intestine by internalizing and degrading the iron exporter, *ferroportin (FPN)*, at the duodenal surface [31]. It blocks the flow of iron into the bloodstream from the iron storage cells and recycling within macrophages (Figure 2) [34]. Additionally, *hepcidin* is highly dependent on *transferrin receptor 2 (TfR2)* and *hereditary hemochromatosis protein (HFE)*, a human homeostatic iron regulator protein encoded by the *HFE* gene [35]. A mutation or deficiency in *TfR2* and *HFE* results in hepcidin deficiency, leading to increased iron absorption or enhanced iron release from macrophages [35].

The frequent measurement of iron is vital for the effective management of β-thalassemia [30]. Although direct estimation of liver iron concentration is the most accurate method to define iron overload in patients, it remains an aggressive procedure [30]. Thus, there is a dire need for a noninvasive method to accurately measure iron storage in the body [30]. Iron overload in the body can be measured using biochemical parameters, such as serum ferritin, hepatic liver concentration, urinary iron excretion, and total iron-binding capacity (TIBC) [36]. However, these parameters give variable results and are inaccurate for iron overload reflection. Currently, serum ferritin estimation is considered the most suitable to reflect iron storage in the body [30]; recently, magnetic resonance imaging (MRI) estimates tissue iron concentration indirectly by detecting the paramagnetic influences of stored iron [37].

**Figure 3:**
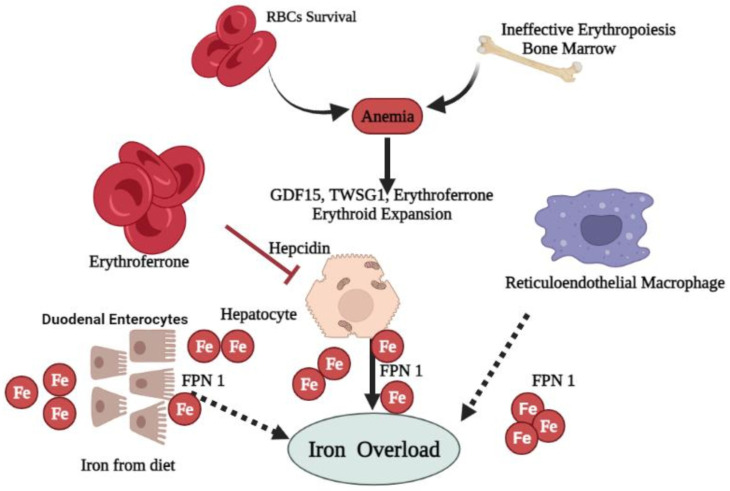
Mechanism of iron dysregulation in β-thalassemia syndrome. Affected patients experience anemia because of IE and shortened red blood cell (RBCs) survival. This condition induces erythropoietin production, leading to enhanced erythropoiesis. The dramatic increase in erythroid expansion activates the erythroid factors, including GDF15, TWSG1, and ERFE secretion. Excessive erythroid factors suppress *hepcidin* expression in liver cells, resulting in iron overload due to increased iron absorption from duodenal enterocytes, an increase in iron from hepatocytes and the reticuloendothelial system. **➔:** resulting in and **⊥:** suppresses expression.

According to research in India, 87.4% of 72 patients of β-thalassemia major and intermedia showed a very high ferritin level due to poor iron chelation, which makes patients more susceptible to iron overload complications [30]. The first dysregulation of *hepcidin* in β-thalassemia was reported using a mouse model [38]. Iron overload is proven to be less dominant in controlling the *hepcidin* expression compared to IE (Figure 3) [38]. Another study revealed that mice with β-thalassemia major and intermedia showed IE and iron deposition within organs, which is associated with suppressed expression of *hepcidin* and an increased level of *FPN* [39]. Even without blood transfusion, 63.8% of HbE/β-thalassemia patients are proven to develop iron overload with serum ferritin (200 to 400 ng/mL) [40,41].

#### 3.2.1. Toxicity Effect of Iron Overload

Excessive iron absorption will result in iron accumulation, damaging vital organs, such as the liver and heart [25]. Iron is a pro-oxidant that induces oxidative stress, contributing to lipid peroxidation, atherosclerosis, DNA damage, carcinogenesis, and neurodegenerative diseases [12]. Moreover, the iron level in the body needs to be controlled for the benefit of resistance towards infection [42]. Bacteria grow faster and form biofilms more readily when iron increases in the body [42]. Therefore, patients with iron overload will be more susceptible to a wide range of intracellular and blood pathogens [42]. Based on a study conducted in Thailand, women with HbE/β-thalassemia demonstrated lower iron usage but higher iron absorption than controls [43]. In HbE/β-thalassemia patients, iron overload is a major problem, requiring regular blood transfusion for their survival [10]. Thus, therapeutic iron chelation therapy is vital in reducing the high iron level [44]. Nevertheless, iron chelators cause severe side effects, such as nausea, diarrhea, dizziness, and elevated liver enzymes, gastrointestinal disorders, and arthralgia [45].

#### 3.2.2. Pathophysiology of Iron Overload in β-Thalassemia

The capacity of the transferrin iron transport system is saturated in β-thalassemia patients, causing the non-transferrin bound iron (NTBI) and labile plasma iron (LPI) to circulate in plasma and eventually be deposited into susceptible cells [30]. The NTBI enters cells through different cellular channels, such as the L-type voltage-dependent Ca^2+^ channel (LVDCC), a promiscuous divalent cation transporter [46], and Zip14, a member of the SLC39A zinc transporter family [47]. Increased iron and labile cellular iron storage result from long-term uptake and accumulation of the NTBI and labile iron pool (LIP) [48], affecting the heart, liver, and endocrine system [49].

When the cellular labile iron pool exceeds the cell ability to synthesize new ferritin molecules, a critical concentration of reactive oxygen species (ROS) is reached. The production of ROS by the NTBI metabolism plays a crucial role in cellular dysfunction, apoptosis, and necrosis [50]. Various ROS, particularly hydroxyl radicals, enhance lipid peroxidation and organelle damage, resulting in cell death [51] and fibrogenesis mediated by transforming growth factor β1 (TGF-β1) [52,53]. Apart from that, iron overload increases the risk of infection, a major cause of death in β-thalassemia patients [54,55]. Autophagy is crucial in eliminating oxidized proteins and damaged mitochondria. Its activation is higher in erythroblasts of HbE/β-thalassemia patients compared to normal control erythroblasts [56]. ROS may promote a higher level of autophagy in HbE/β-thalassemia erythroblasts, thus intensifying apoptosis and IE in HbE/β-thalassemia patients [56]. HbE/β-thalassemia patients with iron overload demonstrate decreased *FPN* expression compared to the healthy control [57]. Excess iron can contribute to IE (Figure 3). Growth differentiation factor 15 (GDF15) and twisted-gastrulation 1 (TWSG1) protein have been reported to suppress *hepcidin* synthesis and enhance iron absorption in β-thalassemia patients [58,59,60]. 

## 4. Hepcidin Expression in β-Thalassemia

*Hepcidin* expression in thalassemia was first reported in a mouse model of severe anemia (C57BI/6 Hbbth3/+) [38]. Furthermore, a decline in serum hepcidin levels has been reported in HbE/β-thalassemia patients, β-thalassemia trait, and HbE trait carriers [61]. The decreased serum hepcidin levels in β-thalassemia patients are associated with the downregulation of *hepcidin* expression in liver cells, resulting in continuous absorption of dietary iron that leads to iron overload [58]. In individuals with thalassemia major and intermedia, liver *hepcidin* mRNA expression is inversely associated with *soluble transferrin receptor (sTfR)* and erythropoietin (EPO), but not with iron storage [62]. Suppression of *hepcidin* in HbE/β-thalassemia patients is linked to increased iron loading, saturated iron-binding proteins, and organ damage [61]. Moreover, *hepcidin* suppression with enhanced iron absorption was found in the β-thalassemia trait [63]. 

### 4.1. Hepcidin Regulation in β-Thalassemia

*Hepcidin* is suppressed in β-thalassemia patients with increasing iron absorption in response to the iron demand by erythroblasts due to tissue hypoxia EPO production and anemia [64]. During the differentiation process, several hepcidin inhibitors are released from erythroblasts to regulate *hepcidin* expression in β-thalassemia. GDF15 serum level is inversely correlated with *hepcidin* expression in hepatocytes of thalassemia patients [65]. Meanwhile, TWSG1 was upregulated in the bone marrow, spleen, and liver of mice with β-thalassemia major and intermedia, associated with *hepcidin* suppression and absence of bone morphogenetic protein (BMP). Additionally, human hepatocytes’ TWSG1 indirectly suppressed *hepcidin* expression through inhibition of BMP-mediated signaling [66].

Erythroferrone (ERFE) hormone functions as a negative regulator of *hepcidin* synthesis. Elevated ERFE expression is associated with increased erythropoietin and *hepcidin* suppression in mice models with thalassemia intermediate during stress erythropoiesis. The ERFE-deficient mice failed to suppress *hepcidin* after hemorrhage and erythropoietin administration [67]. Therefore, increased iron absorption in β-thalassemia is most likely attributed to increased ERFE expression and other hypoxia-related molecules that suppress *hepcidin* synthesis or increase *FPN* expression [39,68].

### 4.2. Regulatory Effect of Hepcidin Transcription

*Hepcidin* is regulated by various stimuli, such as inflammation, plasma iron, anemia, and hypoxia. Its expression is inversely correlated with serum ferritin and induced by iron loading and inflammation. *Hepcidin* dysregulation is the underlying cause of several iron disorders. Erythropoietic activity is the main regulator of *hepcidin* transcription by stimulating erythropoiesis and increasing iron absorption via *hepcidin* downregulation. Chromatin immunoprecipitation analysis showed that the binding of CCAT enhancer binding protein (C/EBPa) to the hepcidin promoter was reduced after EPO supplementation. This indicates C/EBPa effects on *hepcidin* transcription in response to erythropoiesis stimulation [69]. Apart from that, erythropoietin levels increased under hypoxic conditions, involving hypoxia-inducible factor (HIF) in *hepcidin* regulation [58,66]. Furthermore, higher erythropoiesis activity and GDF15 are responsible for low hepcidin levels instead of high EPO levels [70].

GDF15, TWSG1, and ERFE have been reported as suppressors of *hepcidin* in β-thalassemia and other iron-containing anemia [58]. GDF15 was initially thought to be a macrophage inhibitory cytokine but it was later proven that its increase indirectly contributes to iron overload in cancer patients and those with sideropenic anemia by downregulating *hepcidin* expression and increasing iron absorption [71,72]. The tumor suppressor p53 drives GDF15, and its expression in the human body increases under stressful conditions, such as hypoxia, cancer, and tissue damage [73,74]. In addition, pregnancy is associated with low serum hepcidin levels in animal models and humans [75], which positively correlates with GDF15. In contrast, *hepcidin* is negatively correlated with EPO and *hemojuvelin (HJV)* during pregnancy [76]. Mutant *TFR2* and *HJV* were associated with *hepcidin* suppression after hemorrhage and high levels of ERFE mRNA in the th3/+ β-thalassemia mouse model. The significance of ERFE needs to be further evaluated in different conditions of IE and iron loading anemia [77,78].

TWSG1 is higher in immature red cell precursors and mice with β-thalassemia. This erythrokine inhibits *hepcidin* transcription by inhibiting the BMP 2/4 pathway of SMAD 1/5/8 phosphorylation [66]. Atonal basic helix–loop–helix (bHLH) transcription factor 8 (ATOH8) has been identified as a candidate for activation of liver *hepcidin* transcription [79]. Hypoxia, hemolysis, hypotransferrinemia, and erythropoietin treatment enhanced erythropoiesis activity and decreased ATOH8 levels in mice. However, erythropoiesis inhibitors increased ATOH8 levels, suggesting the interference between erythropoiesis and *hepcidin* regulation [79].

Inflammatory cytokines mainly induce *hepcidin* transcription by activating the STAT3 signaling pathway [80]. The BMP–SMAD signaling pathway also plays an essential role in regulating *hepcidin* transcription. Binding of BMPs (BMP2,4,5,6) to type I or type II serine or threonine kinase receptors leads to intracellular R-SMADS (SMAD1, 5 and 8) phosphorylation, which, in turn, binds to SMAD4 (Co-SMAD) to promote its nucleus translocation, thus activating the *hepcidin* transcription. Furthermore, iron management in the body activates BMP/SMAD and *hepcidin* signaling [81]. Andriopoulos et al. reported that BMP6 physically interacts with *HJV* and induces *hepcidin* to lower serum iron in mice [82]. *HFE* is also involved in *hepcidin* pathway regulation [83,84]. Mutations in *HFE* genes involved in the regulation of iron homeostasis cause type I hereditary hemochromatosis (HH) [85]. Additionally, an *HFE*-deficient mouse develops an iron overload phenotype similar to type I HH in humans [84]. These findings suggest that *HFE* positively modulates *hepcidin* expression [86]. Besides, *HFE* interacts with *transferrin receptor 1 (TfR1)* and contends with the receptor’s transferrin (Tf) binding site [87], resulting in the activation of downstream signaling pathways, such as the mitogen-activated protein kinase (MAPK) pathway [81]. Moreover, the crosstalk between the activated MAPK pathway and the BMP/SMAD pathway enhances *hepcidin* expression [88]. 

BMP6 expression is positively associated with the liver iron [89], and its binding to the BMP receptor activates SMAD1/5/8 phosphorylation and upregulates *hepcidin* expression. *HJV* is required for the activation of the BMP/SMAD pathway [90,91]. *TfR2* interacts with BMP and *HJV*, which induces TfR2/HFE complex and BMP signaling, resulting in *hepcidin* expression [92]. USF1/USF2 are involved in *hepcidin* transcription by interacting with E-boxes present in the hepcidin promoter [93]. Many pathways, such as Ras/Raf, MAPK, and mammalian target of rapamycin (mTOR) signaling pathways, are closely related to the regulation of *hepcidin* expression [94]. The *hepcidin*-mediated autoregulation pathway is bound to STAT and inhibits *hepcidin* expression [95]. The mechanism of *hepcidin* inhibition during iron deficiency was elucidated in mice. Phenotypic characteristics include the gradual loss of body hair with microcytic anemia and high hepcidin levels, leading to reduced iron absorption [96]. TMPRSS6 plays a critical role for regulating iron metabolism and iron homeostasis. It interacts with *HJV* and BMP/SMAD signaling to regulate the *hepcidin* expression [97].

### 4.3. Hepcidin Therapeutics in β-Thalassemia

The current treatment of iron overload in β-thalassemia patients includes the administration of iron chelators, such as deferiprone, deferasirox, and desferrioxamine [98,99]. Chelation therapy is recommended in patients with serum ferritin greater than 1000 ng/mL [100]. The direct scavenging of LPI and NTBI by chelators helps prevent adverse sequelae of iron overload [101]. On the other hand, splenectomy has been recommended when the transfusion requirement increases and worsens anemia [102]. Besides, the allogenic hematopoietic stem cell transplantation can also be a therapeutic option for hereditary β-thalassemia, but 60% of patients lack suitable donors, thus increasing the risk of developing transplant-related complications [103].

The correlation between iron overload and hepcidin has led to new approaches that target the disease pathophysiology, aiming to reduce iron overload and IE [104]. A previous study on β-thalassemia mice indicated that a rise in hepcidin level lowers iron bioavailable to erythroblasts, resulting in decreased heme synthesis and improved erythroid precursor and reticulocyte survival [105]. Furthermore, decreasing hepcidin levels in thalassemia leads to iron overload and restores hepcidin to normal and, hence, is a novel therapeutic approach for thalassemia patients [61]. The ligand of the BMP6 receptor is involved in *hepcidin* regulation and transcription [106]. Meanwhile, transferrin is a limiting factor and restricts iron availability for erythropoiesis [107]. TMPRSS6 is a negative regulator of *hepcidin*, and its depletion using small interfering ribonucleic acid siRNA increased *hepcidin* mRNA and improved erythropoiesis in a β-thalassemia mouse model [108]. Furthermore, the SiRNA therapy decreases TMPRSS6 expression, thus increasing *hepcidin* expression and improving the incidence of disease-related thalassemia [109]. Moreover, it is reported that the combined administration of iron chelator deferiprone for RNAi targeting TMPRSS6 can significantly reduce iron content in the liver and increase the efficiency of erythropoiesis in β-thalassemia mice [101,110].

Fibroblast growth factor 23 (FGF23) is a recently discovered hormone that regulates calcium (Ca) and phosphate (P) metabolism [111,112]. It is a 251-amino-acid protein with a molecular weight of 26KDa that is synthesized and secreted by osteoblasts [113,114]. Several studies have emphasized the interaction between iron (Fe) and FGF23 [115,116]. *Hepcidin* binds to *FPN* and internalizes to destroy *FPN* in the proteasome [117,118]. In HbE/β-thalassemia patients, serum levels of FGF23 are incredibly high [119]. Therefore, the direct effect of human FGF23 on the expression of *hepcidin* and *FPN* in HepG2 cells was investigated, demonstrating that the upregulation of *hepcidin* was associated with a significant *FPN* downregulation [120]. Thus, FGF23 expression is considered a key regulator of *hepcidin* expression [121,122]. 

## 5. Signaling Pathways

### 5.1. JAK/STAT Signaling Pathway

**Figure 4:**
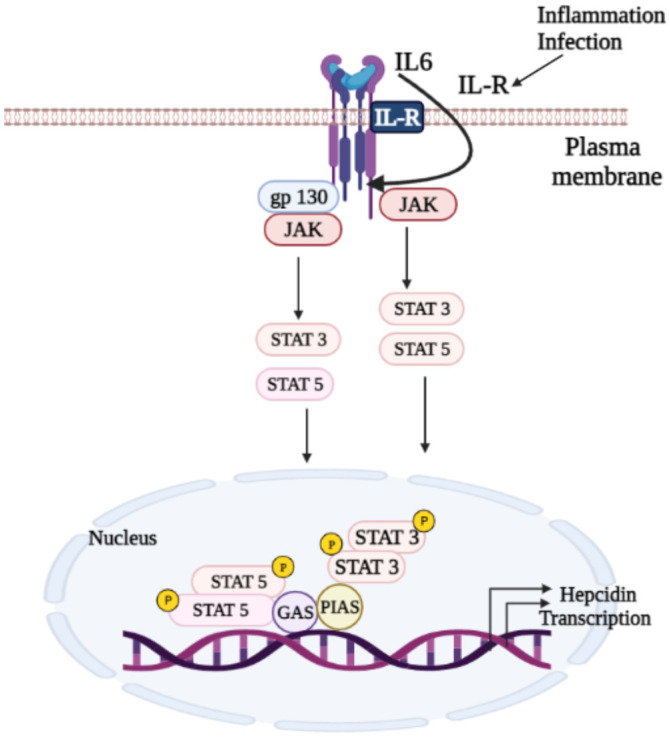
Several regulatory pathways, including JAK/STAT in *hepcidin* transcription. The activation of JAKs after ligand–receptor coupling stimulates phosphorylation of STATs, followed by STAT dimerization and nucleus translocation to activate *hepcidin* transcription. **➔:** Activation/Nucleus translocation.

The JAK/STAT signaling pathway is one of the most important signaling cascades that regulates various cellular biological activities, including cell growth, differentiation, and hematopoiesis [79,123]. There are four members of the JAK family: JAK1, JAK2, JAK3, and tyrosine kinase 2 (TYK2) [124,125]. JAKs activate their downstream targets, STATs, a family of transcription factors consisting of seven members in mammals: STATs 1–4, STAT5A, STAT5B, and STAT6 [124]. Phosphorylation of STAT by JAK or Src kinases give rise to STAT dimerization and nuclear translocation to enhance target gene transcription (see Figure 4) [126].

#### Biological Roles of JAK/STAT Signaling Pathway

Many studies have reported that JAK/STAT signaling is aberrantly activated in hepatocellular carcinoma (HCC), dysregulating their downstream target genes associated with proliferation, immune, invasion, and metastasis [127,128]. *Hepcidin* transcription is regulated by BMP/SMAD and JAK/STAT pathways in response to inflammatory mediators and erythropoietic pathways [129,130]. The JAK/STAT signaling pathway is crucial in inflammation-induced *hepcidin* expression [127]. The discovery of JAK2 as a vital mediator of IE in β-thalassemia suggests that the use of small organic molecules, such as desferrioxamine (DFO) and deferasirox (DFX) chelators, for iron depletion in HH patients and JAK2 inhibition may reduce IE [131]. The reduced erythropoiesis indirectly increases serum hepcidin, reducing intestinal iron absorption and overload [39]. 

## 6. TGF-β/SMAD Signaling

SMADs are proteins that are activated by the transforming growth factor β (TGF-β), BMP signaling, to mediate cell proliferation and differentiation [132,133]. Endosome-associated Fab1 (yeast orthologue of PIKfyve, YOTB, vesicle transport protein (Vac1), and EEA1 (FYVE zinc finger domain)-domain protein (endofin)) influences hepcidin expression by regulating SMAD1/5/8 phosphorylation [134]. STAT and SMAD signaling regulate *hepcidin* expression [135]. Ablation of SMAD4, specifically in the liver, triggers an iron overload in multiple organs due to decreased levels of liver hepcidin [136]. However, SMAD7 acts as an effective inhibitor of *hepcidin* mRNA expression through a negative regulation effect on TGF-β and BMP/SMAD signaling [137,138]. TGF-β is the prototypical ligand of the TGF-β superfamily, which signals during activation of serine/threonine receptor kinases. This superfamily is subdivided into the TGFβ/activin branch and BMP/growth and differentiation factor (GDF) branch.

TGF-β is expressed in most cell types and translated into a proprotein that is proteolytically cleaved into a noncovalently linked mature TGF-β and latency-associated protein (LAP) [139,140]. The active TGF-β ligand is a 25 kDa dimer, covalently linked by bisulfide bonds between cysteine residues of each monomeric peptide [139,140]. 

Various mechanisms are used to regulate the bioavailability of TGF-β in vivo. Once the bioavailable TGF-β reaches the target cell’s surface, it bonds with the homodimer of TGF-β type II receptor (TβRII) [141]. The TGF-β–TβRII complex provides a structural interface that forms a stable complex with the homodimer of the TGF-β type I receptor (TβRI) [132]. Subsequently, the active receptor–ligand complex is a heterotetrametric complex composed of TGFβ dimer and homodimer of TβRII and TβRI. In the active receptor complex, TβRII is constitutively activated and stimulates the transphosphorylation of TβRI [132,142]. In the TGF-β pathway, SMAD2 and SMAD3 are receptor-regulated effector proteins (R-SMADs) phosphorylated by activated TβRI on the C-terminal SSXS motif, leading to the nuclear accumulation of R-SMAD [132].

The activated receptor complex bound to the ligand is internalized by endocytosis [143]. Internalization of cell surface receptors can occur through clathrin-mediated or caveolae-mediated endocytosis [144].

**Figure 5:**
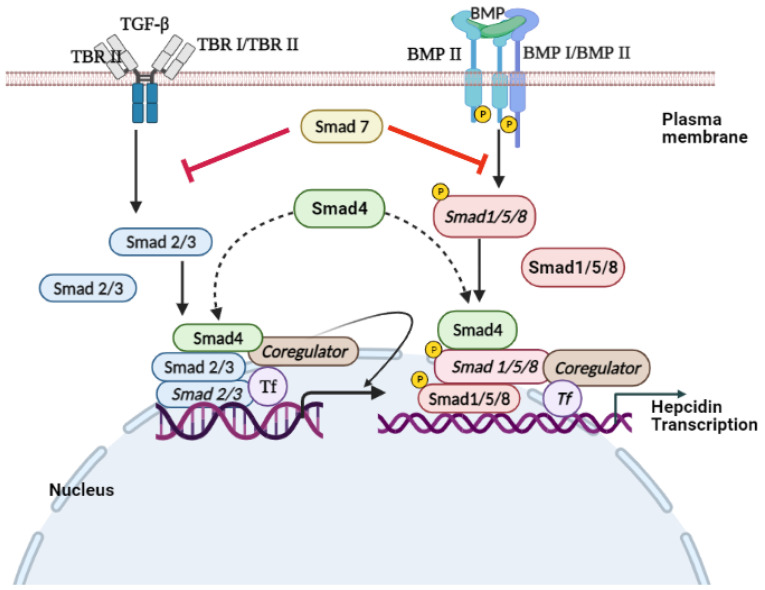
Signal transduction of TGF-β and BMP. The binding of TGF-β to the TβRII dimer allows the ligand to bind to the TβRI dimer and stimulate TβRI kinase activity. In SMAD-mediated TGF-β signal transduction, TβRI phosphorylates cytoplasmic SMAD2 and SMAD3, which interact with SMAD4 after dissociating from TβRI. The two receptors activate the trimeric complex of SMAD2 and SMAD3, and a SMAD4 then enters the nucleus, where it interacts with the DNA-binding transcription factor (TF) and coregulators of the target gene. Similarly, the BMP signals run parallel to the TGF-β signals. In response to the binding of the BMP ligand to the BMPRII heteromeric receptor complex and BMPRI transmembrane kinase, receptor-activated SMAD1 and SMAD5 bind to SMAD4 and are transported to the nucleus to activate or inhibit transcription of *hepcidin*. **➔:** Activation and **⊥:** Inhibit.

Upon ligand stimulation, the SMADs accumulate in the nucleus as R-SMAD/CO-SMAD complex, leading to a decrease in their nuclear export rate [145,146]. The SMAD complex binds to DNA with other transcription factors and interacts with the general transcription machinery to regulate the expression of target genes (Figure 5) [147]. 

Signaling pathways such as STAT and SMAD regulate the expression of *hepcidin*. Therefore, it is hypothesized that STAT and SMAD could be dephosphorylated in thalassemia cases, including HbE/β-thalassemia patients, resulting in *hepcidin* downregulation with the presence of iron accumulation.

## 7. Conclusions

**Figure 6:**
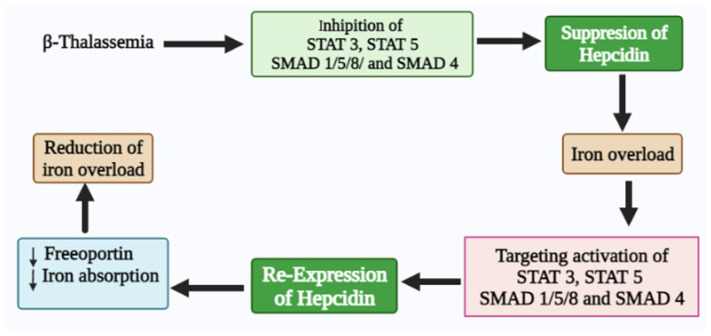
Regulation of STAT and SMAD signaling pathway on hepcidin expression.

In summary, iron homeostasis dysregulation has a dominant physiological effect on transfusion-dependent and transfusion-independent β-thalassemia patients. Thus, understanding the expression of *hepcidin* and its regulation in β-thalassemia patients is vital in developing rational therapeutic interventions to provide safe, effective, and lifelong treatment options for their management. Therefore, recovery of *hepcidin* in β-thalassemia patients through the activation of STAT 3, STAT 5, SMAD 1/5/8, and SMAD 4 signaling could be a potential therapeutic target for managing iron overload (Figure 6). Therefore, it is highly recommended for future preclinical and clinical studies to evaluate the related risks and benefits of *hepcidin*-targeted treatment approaches.

## Data Availability

Not applicable.
